# Large multi‐centre pilot randomized controlled trial testing a low‐cost, tailored, self‐help smoking cessation text message intervention for pregnant smokers (MiQuit)

**DOI:** 10.1111/add.13802

**Published:** 2017-05-02

**Authors:** Felix Naughton, Sue Cooper, Katharine Foster, Joanne Emery, Jo Leonardi‐Bee, Stephen Sutton, Matthew Jones, Michael Ussher, Rachel Whitemore, Matthew Leighton, Alan Montgomery, Steve Parrott, Tim Coleman

**Affiliations:** ^1^ School of Health SciencesUniversity of East AngliaNorwichUK; ^2^ Division of Primary CareUniversity of NottinghamNottinghamUK; ^3^ Behavioural Science GroupUniversity of CambridgeCambridgeUK; ^4^ Division of Epidemiology and Public HealthUniversity of NottinghamNottinghamUK; ^5^ Population Health Research InstituteSt George's University of LondonLondonUK; ^6^ Nottingham Clinical Trials UnitUniversity of NottinghamNottinghamUK; ^7^ Department of Health SciencesUniversity of YorkYorkUK; ^8^ UK Centre for Tobacco and Alcohol StudiesUniversity of NottinghamNottinghamUK

**Keywords:** mHealth, pregnancy, randomized controlled trial, self‐help, smoking cessation, SMS text messaging

## Abstract

**Aims:**

To estimate the effectiveness of pregnancy smoking cessation support delivered by short message service (SMS) text message and key parameters needed to plan a definitive trial.

**Design:**

Multi‐centre, parallel‐group, single‐blinded, individual randomized controlled trial.

**Setting:**

Sixteen antenatal clinics in England.

**Participants:**

Four hundred and seven participants were randomized to the intervention (*n* = 203) or usual care (*n* = 204). Eligible women were < 25 weeks gestation, smoked at least one daily cigarette (> 5 pre‐pregnancy), were able to receive and understand English SMS texts and were not already using text‐based cessation support.

**Intervention:**

All participants received a smoking cessation leaflet; intervention participants also received a 12‐week programme of individually tailored, automated, interactive, self‐help smoking cessation text messages (MiQuit).

**Outcome measurements:**

Seven smoking outcomes, including validated continuous abstinence from 4 weeks post‐randomization until 36 weeks gestation, design parameters for a future trial and cost‐per‐quitter.

**Findings:**

Using the validated, continuous abstinence outcome, 5.4% (11 of 203) of MiQuit participants were abstinent versus 2.0% (four of 204) of usual care participants [odds ratio (OR) = 2.7, 95% confidence interval (CI) = 0.93–9.35]. The Bayes factor for this outcome was 2.23. Completeness of follow‐up at 36 weeks gestation was similar in both groups; provision of self‐report smoking data was 64% (MiQuit) and 65% (usual care) and abstinence validation rates were 56% (MiQuit) and 61% (usual care). The incremental cost‐per‐quitter was £133.53 (95% CI = –£395.78 to 843.62).

**Conclusions:**

There was some evidence, although not conclusive, that a text‐messaging programme may increase cessation rates in pregnant smokers when provided alongside routine NHS cessation care.

## Introduction

Smoking in pregnancy is associated strongly with pregnancy complications, including miscarriage 1, spontaneous preterm birth 2, small for gestational age 2 and stillbirth 3, 4. Smoking in pregnancy also perpetuates health inequalities; rates are approximately five times higher in the most deprived women compared with the least deprived 5, 6, 7 and children born to smokers have an increased risk of becoming smokers themselves 8, 9. Systematic review evidence shows that behavioural smoking cessation interventions reduce the risks of preterm birth and low birth weight by approximately 18% 10.

Structured self‐help support helps pregnant smokers to stop 11, 12. Mobile phone text messaging is a simple way of providing self‐help support and is effective for non‐pregnant smokers 13. However, many aspects of ‘generic’ text messaging cessation systems are unlikely to be appropriate in pregnancy. Available generic programmes make no mention of pregnancy 13, which for most pregnant smokers is the main reason they try quitting 14, and effective behavioural support for pregnant smokers is typically strongly pregnancy‐orientated 10. Consequently, pregnant smokers may find much of the behavioural support delivered by generic programmes irrelevant, reducing its impact and perhaps even being counterproductive 15. Even more importantly, available ‘generic’ programmes provide some advice and support that potentially could be harmful in pregnancy. For example, use of nicotine replacement therapy (NRT) is encouraged without consideration of pregnancy‐specific risks, and generic advice on keeping fit and weight gain after quitting are quite different from what might be appropriate in pregnancy.

To maximize the potential of self‐help support for helping pregnant smokers to stop, we have developed an individually tailored short message service (SMS) text messaging intervention specifically for pregnant smokers, called MiQuit. This process followed the Medical Research Council framework for developing and evaluating complex interventions 16 and was informed by extensive qualitative work with pregnant smokers 17. MiQuit can be used by all pregnant smokers, as the support it provides is tailored to a woman's level of motivation to quit. A randomized controlled trial (RCT) (*n* = 207) demonstrated that randomization to MiQuit or routine care is feasible, that women find MiQuit highly acceptable and that MiQuit is likely to encourage cessation until at least mid‐pregnancy 18. This feasibility trial provided the best available estimate for MiQuit efficacy, albeit for a relatively brief cessation period; we believed cessation at the end of pregnancy would be a more appropriate outcome for a definitive trial, as this would result in maximal benefits for the fetus. As MiQuit is a cheap intervention with potential for wide dissemination, we anticipated that even a 1–2% absolute effect on smoking cessation in pregnancy could prove clinically important and cost‐effective and the imprecise efficacy estimate we had obtained suggested that an impact of this size was potentially attainable. Consequently, we planned a full trial to detect such an effect on smoking cessation until the end of pregnancy, and estimated this could require 3–4000 participants. This large, pilot RCT was conducted to investigate the feasibility of undertaking a much larger multi‐centre RCT in UK National Health Service (NHS) settings to determine whether or not MiQuit can impact upon cessation throughout pregnancy. The current trial would also provide estimates of effectiveness and cost‐effectiveness, with the latter enabling comparisons with other cessation interventions.

## Methods

### Design

This was a multi‐centre, two‐arm, parallel group, single‐blind, individually randomized controlled trial.

### Study population

Participants were recruited from 16 English NHS hospital antenatal clinics between February and September 2014. They were aged 16 years and over, less than 25 weeks pregnant, had smoked at least five cigarettes daily before pregnancy and at least one per day at enrolment, able to understand written English and owned a mobile phone with text messaging functionality. Participants already using text message‐based smoking cessation support were excluded.

### Study protocol and interventions

The study protocol was approved by Nottingham 1 Research Ethics Committee (Ref.:13/EM/0427) and subsequently published 19.

#### Usual care

Participants were given a standard NHS booklet on smoking cessation for mothers‐to‐be (Supporting information, Appendix S1) and could access smoking cessation information, advice or support for stopping smoking offered as part of routine antenatal care.

#### Intervention

Two days after enrolment, in addition to the booklet and usual care, intervention participants started to receive MiQuit: an automated 12‐week advice and support programme for quitting smoking in pregnancy delivered by SMS text message. MiQuit objectives are informed by Social Cognitive Theory 20, Perspectives on Change Theory (Borland, 2000, unpublished work), the Elaboration Likelihood Model of Persuasion 21 and several additional cognitive determinants of quitting smoking in pregnancy 18. It uses 14 participant characteristics to tailor support individually 22. Tailoring characteristics include gestation, motivation to quit, the hardest situation to avoid smoking, cessation self‐efficacy, cigarette dependence and partner's smoking status. ‘Push’ support (i.e. automated support sent to participants’ phones) is delivered according to a delivery schedule (0, 1 or 2 daily texts). Push message frequency is highest in the first 4 weeks. Push support includes motivational messages, advice about quit attempt preparation, managing cravings and withdrawal, dealing with trigger situations and preventing lapses, information about fetal development and how smoking affects this (see Supporting information, Appendix S2 for example messages and tailoring variables). Users can alter support frequency by texting the keywords MORE or LESS, and are encouraged to set and send a quit date to MiQuit to enable them to receive additional support orientated around when their quit attempt begins. At 3 and 7 weeks into the programme, users are asked to respond to texts asking about smoking in the previous 3 days, so that subsequent support is further tailored to smoking behaviour 22. Additionally, system users can ‘pull’ on‐demand support for combatting cravings or temptation to smoke by texting HELP and seek advice on returning to abstinence after a lapse by texting SLIP. Alternatively, texting QUIZ provides a multiple‐choice message trivia game designed to distract users from smoking. Support can be discontinued by texting STOP. More detailed information about the development and structure of the intervention can be found elsewhere [18,22].

#### Enrolment, randomization and blinding

Research midwives (RMs) identified potential participants in antenatal clinics via their clinic notes or a screening questionnaire, and interested women were provided with participant information sheets. RMs sought written consent, but if time was insufficient, contact details were requested instead and verbal consent was sought later in a phone call from the RM or a researcher from the trial coordination team. Next, baseline data were collected and, after this was entered onto a web‐based database, participants were randomized individually to usual care or the MiQuit intervention in a 1 : 1 ratio using the Nottingham Clinical Trials Unit web‐based system, with both the RM or researcher and the participant remaining masked to allocation. Randomization used a computer‐generated pseudo‐random code with random permuted blocks of randomly varying size, and stratification was by study site and gestation (<16 versus ≥16 weeks). Following randomization, unblinded trial team members sent arm‐specific information packs to participants, which included the usual care booklet. Those dispatching packs were not involved in collecting follow‐up data. Trial staff involved in follow‐up remained unaware of participants’ treatments until questions on the intervention were asked at the end of the study, after smoking outcome data had been collected.

#### Data collection

Baseline data included contact details, age, highest qualification, postcode to enable matching to Index of Multiple Deprivation (IMD) scores 23, ethnicity (based on UK Census categories), gestation, pre‐pregnancy smoking rate, heaviness of smoking index 24, strength and frequency of urges to smoke 25, whether a quit date had been set, intention to quit 18, number of births beyond 24 weeks, partner's (significant other's) smoking status and health status using the EuroQol five dimensions questionnaire (EQ‐5D) 26.

Four weeks after randomization, participants were contacted to complete a questionnaire assessing smoking status during the past 7 days; we used text messages to notify them to expect a telephone call and if after several attempts the call was unsuccessful, we posted and e‐mailed a link to the questionnaire. At 36 weeks gestation participants were contacted similarly and asked about smoking behaviour since 4 weeks post‐randomization and in the past 7 days, quit attempts lasting at least 24 hours and use of smoking cessation support. MiQuit arm participants were also asked their views on the intervention. Where 7‐day complete abstinence from smoking was reported, we immediately attempted to validate this biochemically with exhaled‐breath carbon monoxide (CO) readings and/or saliva samples tested for cotinine, with samples or readings collected at hospital or home visits. If face‐to‐face collection was not successful, postal saliva sample packs were used. Before samples were donated, participants were asked either verbally or by questionnaire about smoking status and use of NRT or e‐cigarettes.

To encourage engagement, participants received a £5 shopping voucher for providing data at each of the first three contacts (i.e. £15 maximum); a £10 voucher was also provided after validation visits. Participants were informed of how to withdraw from data collection via postcard, telephone, text or e‐mail.

### Outcomes

#### Future trial design parameters

We monitored monthly rates of recruitment and outcome ascertainment rates, and estimated the validated abstinence rate in both trial arms combined. We aimed to enrol 400 participants in 12 months. The key smoking outcome for a future trial is described below (outcome 1).

#### Smoking

Smoking measures were: (1) self‐reported abstinence from 4 weeks post‐randomization until late pregnancy collected at late pregnancy follow‐up (approximately 36 weeks gestation), with no more than five cigarettes in total between the two time‐points 27, biochemically validated at the later time; (2) as 1 but self‐report only; (3) self‐reported 7‐day point prevalence abstinence at late pregnancy; (4) as 3 but validated biochemically; (5) self‐reported 7‐day point prevalence abstinence at 4 weeks post‐randomization; 6) self‐reported 7‐day point prevalence abstinence at both 4 weeks post‐randomization and late pregnancy; and (7) as 6 but validated biochemically in late pregnancy.

We stated a priori that we anticipated that outcome 1, continuous abstinence from 4 weeks post‐randomization until 36 weeks gestation, would be most appropriate for a future RCT to assess MiQuit efficacy definitively 19. We had concerns about the viability of using this outcome, so a key objective was to ascertain its feasibility of measurement. Where participants reported abstinence but were using NRT or e‐cigarettes, CO readings alone were used for validation [cut‐point of < 9 parts per million (p.p.m.)]. Otherwise, a saliva cotinine reading of < 10 ng/ml was also required 28. Where data from only one validation method were available, a value below the relevant cut‐point was considered sufficient. Saliva was analysed by ABS Laboratories Ltd, Hertfordshire.

#### Economics

As the usual care and intervention groups both had access to standard NHS smoking cessation and antenatal care, it was assumed that both groups had equal cost, therefore the only additional cost would be for delivering MiQuit. Costs included were the text messages and the annual running cost. These were based on historical costs incurred. Costs were calculated at 2014–15 price per year from a NHS and Personal Social Services perspective.

#### Sample size

The sample size was justified primarily on the basis of how precisely key parameters for the design of a definitive RCT could be estimated. With 400 participants (200 per group), we could estimate the overall recruitment rate to within ±1%, outcome ascertainment rates per treatment group to within ±4% and combined quit rates for both groups to within ±3%. Precision estimates for detecting between‐group differences in quit rates were calculated for ranges of treatment effects [i.e. odds ratio (OR)] and usual care group quit rates 19 for example, these showed that if a 5% usual care group quit rate occurred in late pregnancy, with 400 participants the trial would estimate an OR of 1.8 (as noted in a previous review) 12 with 80% confidence intervals (CIs) of 1.06–3.05) 19.

### Statistical analysis

A statistical analysis plan was agreed with the Trial Steering Committee and published with the trial protocol 19. Recruitment and outcome ascertainment rates were estimated with 95% CIs. For each treatment group, and for both groups combined, abstinence rates for each outcome were estimated with 95% Wilson CIs. χ^2^ tests (Fisher's exact tests in cases with small expected frequencies) were performed to assess the association between smoking outcomes and treatment group. Firth (penalized) logistic regression models 29 were then used to estimate ORs with 95% profile CIs 30 to compare smoking outcomes between treatment groups, adjusting for factors used to stratify the randomization via their inclusion as fixed covariates in each model (trial site, gestation at randomization). Three additional models for all seven smoking outcomes were carried out, each adjusting for one of three baseline variables associated commonly with smoking in pregnancy (heaviness of smoking, partner's smoking status and education) 31, 32, with likelihood ratio tests assessing whether these improved model prediction. Where convergence of a model could not be achieved due to low event rates within small centre sites, these centres were merged to overcome the issue.

An intention‐to‐treat (ITT) analysis was used, with all participants analysed within the treatment group to which they were randomized and, where missing outcome data, were assumed smoking 27. Participants who withdrew from the study due to miscarriage/stillbirth were included in the analyses and classed as smoking. Where validation of abstinence was required, participants not providing a breath or saliva sample were classed as smoking. Complete case sensitivity analyses were performed on all smoking outcomes.

The number of quit attempts since baseline was compared between groups using a Mann–Whitney *U*‐test. Participants’ views on the MiQuit intervention were reported using percentages with 95% Wilson score CIs. Analyses were carried out in Stata, version 12.

After undertaking the planned analyses, we decided to generate a Bayes factor from smoking outcome 1, using an online calculator 33 with an expected effect size of OR = 1.83 taken from a relevant systematic review 12. We used a conservative approach for estimation using a half‐normal distribution, where the mode at 0 indicated no intervention effect and the standard deviation equal to the expected effect size.

### Economic analysis

The main outcome was the incremental cost per additional quitter, calculated by dividing the average incremental cost per participant by the number of additional quitters derived from smoking outcome 1. Confidence intervals were generated using bootstrapping with 1000 iterations 34.

## Results

**Figure 1:**
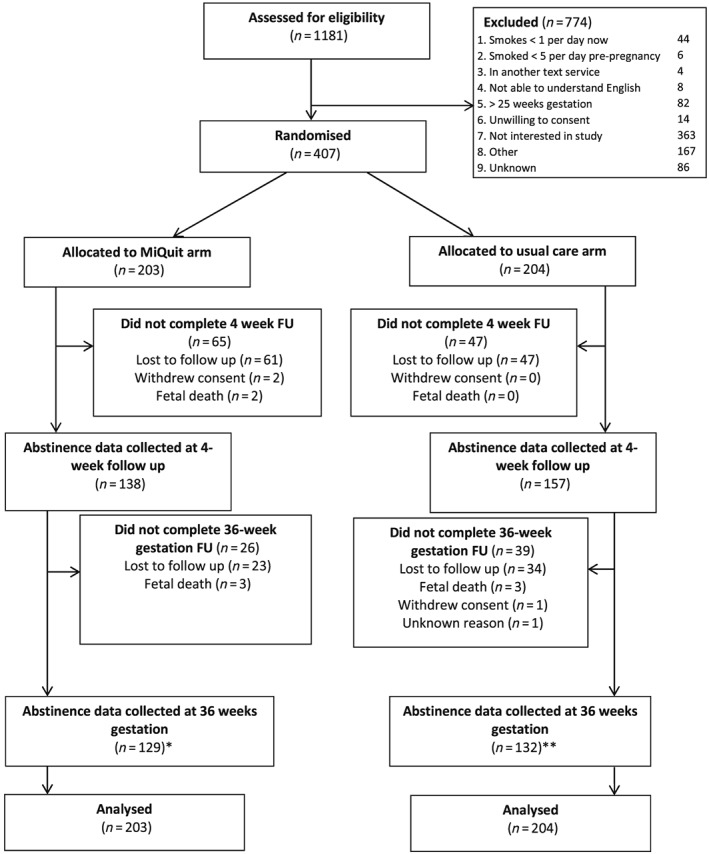
Trial flow. *Includes 17 MiQuit participants without 4‐week follow up data; **includes 14 usual care participants without 4‐week follow‐up data

During 7 months, we assessed 1181 pregnant smokers for eligibility and 407 were recruited into the study; 203 were randomized to MiQuit and 204 to usual care. There was marked variation in recruitment between the 16 sites [median 12 participants, interquartile range (IQR) = 34], with one recruiting no participants. Figure 1 shows participant flow and reasons for exclusion. At 4 weeks, 295 (72%) participants provided smoking outcome data (68% MiQuit, 77% usual care). Further attrition in late pregnancy was fairly minimal, with 261 (64%) participants providing these outcome data (64% MiQuit, 65% usual care). Two hundred and thirty (57%) provided smoking outcome data at both time‐points (55% MiQuit, 58% usual care) and 254 (62%) gave data used for smoking outcome 1 on abstinence between 4 weeks and late pregnancy (61% MiQuit, 64% usual care). We obtained validation samples for 37 of 64 (58%) of participants who reported abstinence at 36 weeks gestation (56% MiQuit, 61% usual care); with two (3.1%) and 15 (23%) participants providing only CO or cotinine readings, respectively.

Table 1 shows baseline participant characteristics by trial arm; mean age was 26.5 [standard deviation (SD) = 5.7] years, 92% were white, mean gestation at enrolment was 14.7 (SD = 4.4) weeks and 60% reported smoking within 30 minutes of waking; 74% were very or extremely determined to stop smoking and 40% felt very or extremely confident in stopping until their baby was born. Participants’ characteristics were similar in both groups, apart from that women randomized into the usual care group were more likely to reside in the most deprived (e.g. lower income) areas and have a non‐smoking partner.

**Table 1:** Baseline characteristics by treatment group.

	MiQuit^b^ (n = 203)	Usual care^b^ (n = 204)
Age (years)		
Mean (SD)	26.6 (5.7)	26.4 (5.7)
Median (1st Q, 3rd Q)	25.7 (22.1, 30.8)	25.8 (21.9, 29.7)
Min, max	16.9, 40.0	16.6, 41.3
Highest qualification		
No formal qualification	37 (18.2)	44 (21.6)
GCSE or similar	117 (57.6)	106 (52.0)
A level or similar	32 (15.8)	37 (18.1)
Degree or similar	16 (7.9)	13 (6.4)
Declined to answer	1 (0.5)	4 (2.0)
IMD score^a^		
Quintile 1	13 (6.4)	6 (2.9)
Quintile 2	16 (7.9)	13 (6.4)
Quintile 3	22 (10.8)	21 (10.3)
Quintile 4	53 (26.1)	50 (24.5)
Quintile 5	92 (45.3)	108 (52.9)
missing	7 (3.5)	6 (2.9)
Ethnicity		
White	188 (92.6)	185 (90.7)
Indian	0 (0)	0 (0)
Pakistani	3 (1.5)	2 (1.0)
Bangladeshi	0 (0)	0 (0)
Black Caribbean	1 (0.5)	4 (2.0)
Black African	2 (1.0)	1 (0.5)
Black (other)	1 (0.5)	1 (0.5)
Chinese	0 (0)	0 (0)
Other Asian (non‐Chinese)	1 (0.5)	0 (0)
Mixed	6 (3.0)	11 (5.4)
Not given	1 (0.5)	0 (0)
Gestation at randomization (weeks)		
Mean (SD)	14.6 (4.2)	14.7 (4.5)
Median (1st Q, 3rd Q)	13 (12, 19)	13 (12, 20)
Min, max	4, 23	3, 24
Cigarettes per day before pregnancy		
Mean (SD)	15.7 (6.7)	16.4 (6.6)
Median (1st Q, 3rd Q)	15 (10, 20)	15 (10, 20)
Min, max	5, 40	5, 40
Cigarettes per day now		
Mean (SD)	9.0 (5.9)	9.4 (6.1)
Median (1st Q, 3rd Q)	8 (5, 10)	10 (5, 10)
Min, max	1, 40	1, 40
Time to first cigarette after waking		
Within 5 minutes	64 (31.5)	64 (31.4)
6–30 minutes	56 (27.6)	61 (29.9)
31–59 minutes	41 (20.2)	31 (15.2)
1–2 hours	22 (10.8)	29 (14.2)
More than 2 hours	20 (9.9)	19 (9.3)
Frequency of urges to smoke in the past 24 hours		
Not at all	3 (1.5)	8 (3.9)
A little of the time	36 (17.7)	37 (18.1)
Some of the time	94 (46.3)	88 (43.1)
A lot of the time	44 (21.7)	42 (20.6)
Almost all the time	16 (7.9)	18 (8.8)
All the time	10 (4.9)	11 (5.4)
Strength of urges to smoke in the past 24 hours		
No urges	4 (2.0)	6 (2.9)
Slight	58 (28.6)	55 (27.0)
Moderate	78 (38.4)	95 (46.6)
Strong	39 (19.2)	28 (13.7)
Very strong	15 (7.4)	14 (6.9)
Extremely strong	9 (4.4)	6 (2.9)
Have you set a quit date?		
No	193 (95.1)	192 (94.1)
Yes	10 (4.9)	12 (5.9)
Are you seriously planning to quit?		
No	17 (8.4)	19 (9.3)
Within the next 3 months	68 (33.5)	57 (27.9)
Within the next 30 days	55 (27.1)	59 (28.9)
Within the next 2 weeks	63 (31.0)	69 (33.8)
Number of births beyond 24 weeks		
Mean (SD)	1.4 (1.5)	1.4 (1.4)
Median (1st Q, 3rd Q)	1 (0, 2)	1 (0, 2)
Min, max	0, 10	0, 9
Parity		
0 births beyond 24 weeks	66 (32.5)	65 (31.9)
1 or more births beyond 24 weeks	137 (67.5)	139 (68.1)
Partner/significan other's smoking status		
Smoker	135 (66.5)	128 (62.8)
Non‐smoker	34 (16.8)	44 (21.6)
Not applicable (no partner)	34 (16.8)	32 (15.7)

Data are *n* (%) unless specified.

a Index of Multiple Deprivation (IMD), Office for National Statistics. Quintile 1 (Q) represents least deprivation.

b Data were complete for all baseline variables other than IMD score (3.2% missing: no match to home postcode), highest qualification (1.2% missing) and ethnicity (0.25% missing). Similar proportions per trial arm were missing baseline data. SD = standard deviation; GCSE = general certificate of standard education; A level =Advanced level.

### Smoking outcomes

Table 2 shows cessation rates across and within treatment groups and provides estimates for MiQuit's effects. For smoking outcome 1, 15 participants were classified as abstinent; 11 of 203 (5.4%) were in the MiQuit group and four of 204 (2.0%) in the usual care group (adjusted OR= 2.70, 95% CI = 0.93–9.35). Estimated treatment effects for the remaining smoking outcomes also favoured MiQuit aiding smoking cessation, with ORs ranging from 1.03 to 3.28; those for self‐reported abstinence at both 4 weeks post‐randomization and in late pregnancy (smoking outcome 6) reached statistical significance. Adjusting for heaviness of smoking, partner's smoking status and education did not result in any meaningful changes to the findings (see Supporting information, Table S1). In a sensitivity analysis based on women with complete outcome data, the ORs were increased for six of the seven smoking outcomes, including outcome 1 (OR = 3.11, 95% CI = 1.05–10.80) (Supporting information, Table S2). The number of quit attempts between baseline and late pregnancy did not differ significantly between treatment groups [MiQuit median 2 (IQR = 1,3), *n* = 124; usual care median 1 (IQR = 0,3), *n* = 130; Mann–Whitney *U*‐test *P* = 0.118). The Bayes factor for outcome 1 was 2.23, meaning that the hypothesis that MiQuit is effective is more than twice as likely to be correct than the hypothesis that it is not effective. This represents ‘anecdotal evidence’ for MiQuit having an intervention effect 35.

**Table 2:** MiQuit treatment effect estimates on seven smoking outcomes.

Outcome	Measure	MiQuit^a^ n = 203 (%)	Usual care^a^ n = 204 (%)	Total^a^ n = 407 (%)	P‐value^b^	Adjusted odds ratio (95% CI)^c^
Abstinence reported from 4 weeks post‐randomization until late pregnancy (smoking outcome 1)^d^	Validated	11 (5.42)	4 (1.96)	15 (3.69)	0.064	2.70 (0.93–9.35)
Abstinence reported from 4 weeks post‐randomization until late pregnancy (smoking outcome 2)^d^	Self‐report	33 (16.26)	33 (16.18)	66 (16.22)	0.983	1.03 (0.61–1.75)
7‐day point prevalence abstinence at late pregnancy (smoking outcome 3)	Self‐report	36 (17.73)	28 (13.73)	64 (15.72)	0.267	1.34 (0.79–2.31)
7‐day point prevalence abstinence at late pregnancy (smoking outcome 4)	Validated	15 (7.39)	9 (4.41)	24 (5.90)	0.202	1.67 (0.72–4.03)
7‐day point prevalence abstinence at 4 weeks post‐randomization (smoking outcome 5)	Self‐report	15 (7.39)	7 (3.43)	22 (5.41)	0.077	2.11 (0.89–5.46)
7‐day point prevalence abstinence at both 4 weeks post‐randomization and late pregnancy (smoking outcome 6)	Self‐report	13 (6.40)	4 (1.96)	17 (4.18)	0.025	3.16 (1.14–10.69)
7‐day point prevalence abstinence at both 4 weeks post‐randomization and late pregnancy (smoking outcome 7)	Validated	8 (3.94)	2 (0.98)	10 (2.46)	0.062	3.28 (0.90–17.36)

a All smoking outcomes are calculated of a total of 407 participants (203 MiQuit, 204 usual care). Participants lost to follow‐up or with missing outcome data are assumed to be smoking.

b Unadjusted, from a χ^2^ test using a two‐sided *P*‐value (Fisher's exact test *P*‐values were used in the case of small expected frequencies).

c Model‐based, adjusted by site and gestation at randomization (95% profile confidence intervals reported).

d Russell standard criterion (permits no more than five cigarettes in total). The criterion for all other smoking outcomes was total abstinence (‘not even a puff’). CI = confidence interval.

### Use of NHS cessation support

Overall use of ‘non‐trial’ cessation support was similar in both arms (Table 3). When examining specific types of support, midwife discussion of smoking was reported by notably more usual care participants.

**Table 3:** Use of National Health Service (NHS) and other cessation support during trial period.

Outcome^a^	MiQuit (n = 124)	Usual care (n = 130)
Reported use of any stop smoking support *n* (%)	83 (66.9)	98 (75.4)
Reported use of different types of support *n* (%)		
GP or nurse discussion	20 (16.1)	26 (20.0)
Midwife discussion	45 (36.3)	72 (55.4)
Stop smoking helpline	5 (4.0)	6 (4.6)
NHS Smokefree website	16 (12.9)	15 (11.5)
Other smoking cessation website	7 (5.7)	9 (6.9)
NRT	26 (21.0)	36 (27.7)
Individual NHS behavioural support	9 (7.3)	15 (11.5)
Group NHS behavioural support	3 (2.4)	3 (2.3)

a Outcomes are calculated out of 254 participants with response data at late pregnancy follow‐up (124 MiQuit, 130 usual care). NRT = nicotine replacement therapy; GP = general practitioner.

### Participant evaluations of MiQuit

Among all MiQuit participants, 27 (13%) discontinued support early (mean days into programme 24.1, SD = 15.7) having texted STOP and 13 (6.4%) changed their message frequency to ‘less’, 11 (5.4%) to ‘more’ and 1 (0.5%) to ‘less’ followed by ‘more’. Among those at late‐pregnancy follow‐up who answered the relevant questions, three of 123 (2.4%) reported receiving no text messages and, of the remaining 120, 97 (81%) reported reading all messages at least once. Messages relating to fetal development were rated most frequently (by 35%) as the most helpful. Table 4 shows that 62% rated the text messages as quite or extremely helpful, but 14% considered them annoying; 81% would either ‘probably’ or ‘definitely’ recommend MiQuit support to a friend or relative.

**Table 4:** Intervention participant views of and preferences for the MiQuit intervention.

	MiQuit
Reported receiving text messages (*n* = 123)	120 (97.6, 93.1–99.2)
Discontinued the support prematurely by texting ‘STOP’ (*n* = 203)	27 (13.3, 9.3–18.7)
Rated the text messages as ‘quite’ or ‘extremely’ helpful (*n* = 120)	74 (61.7, 52.7–69.9)
Rated the text messages as ‘quite or ‘extremely’ annoying (*n* = 120)	17 (14.2, 9.0–21.5)
Rated the number of text messages received as (*n* = 120)	
‘Far too many’ or ‘a little too many’	25 (20.8, 14.4–29.2)
‘About right’	79 (65.8, 56.8–73.9)
‘Not enough’ or ‘not nearly enough’	16 (13.3, 8.3–20.8)
Would ‘probably’ or ‘definitely’ recommend the support (*n* = 120)	97 (80.8, 72.8–86.9)

Data are *n* %, 95% Wilson confidence interval (CI).

### Economic analysis

The per‐participant cost of sending MiQuit texts was estimated to be £2.95; a mean of 84.1 texts per participant at 3.5 p per text. The annual running cost of delivering MiQuit was £339 (£1.67 per participant) and included a virtual reply number (£99) and server/web hosting including domain name (£240). Thus, the total per‐participant MiQuit cost was £4.62. From Table 2, row 1, the relevant incremental quit rate estimate was 3.46%, giving an incremental cost per additional quitter of £133.53 (95% CI = –£395.78 to 843.62). The probability of MiQuit being cost‐effective was 96.5% if a decision‐maker was willing to pay £10 000 to gain an additional quitter.

## Discussion

### Statement of principal findings

This trial demonstrates the feasibility of recruiting pregnant smokers from multiple UK hospital antenatal settings to a trial of a text message cessation support intervention; we met our recruitment target 5 months earlier than expected. We also found that it was feasible to measure smoking cessation in participants who were not expected to set a quit date using a stringent outcome measure. Using this outcome, we found that 5.4% of women in the MiQuit group stopped smoking during pregnancy and 2.0% did in the control group, and this almost reached statistical significance; it is likely, when tested on a larger scale, that MiQuit will prove to be both effective and cost‐effective for promoting smoking cessation throughout pregnancy.

### Findings in context

The efficacy estimate provided using outcome 1 data suggests that, compared with usual care, MiQuit may almost triple the odds of sustained smoking cessation, but this has limited precision. However, it is the best estimate yet produced for the probable efficacy of text messaging used for smoking cessation in pregnancy. It is also of a similar magnitude to efficacy estimates derived from ‘definitive’ trials of similar interventions used by non‐pregnant smokers 36, 37 and to that from a smaller MiQuit trial 18. Additionally, our estimate for the probable cost‐effectiveness of the intervention is encouraging; compared with other cessation interventions, a cost‐per‐quitter of £134 is low. For example, although financial incentives for smoking cessation in pregnancy are highly effective 38 and cost‐effective 39, their cost‐per‐quitter is almost 10 times higher (£1127). Similarly, MiQuit's cost‐efficacy compares favourably with that of cessation support delivered by traditional UK smoking cessation services; the ‘cost‐per‐person‐setting‐a‐quit‐date’ within such services has been estimated recently as £202 40. However, as only 34% of those setting a quit date achieve longer‐term abstinence, the cost‐per‐quitter 41, inflated accordingly, is probably closer to £600. Although the trial did not include a formal cost–utility analysis, it is highly likely that, if cessation is maintained in the longer term, the calculated ‘cost‐per‐quitter’ will translate into longer‐term cost‐effectiveness. One can assume that ‘quitters’ gain 1.94 quality‐adjusted life years (QALYs) across their life‐time 42, 43, so by multiplying this value by the seven additional quitters generated by MiQuit the incremental QALYs would be 13.58, making the incremental cost per additional QALY £69.06—even after inflating this figure to take into account relapse to smoking 44, this would remain securely within most accepted cost‐effectiveness benchmarks. Finally, it is noteworthy that the ‘non‐text‐message’ costs of MiQuit are fixed, and so ‘per‐user’ costs fall as the numbers using the intervention increase. For example, if MiQuit was used by 2000 pregnant smokers annually, per‐user ‘non‐text‐message’ costs would be approximately £0.20, reducing the incremental cost per additional quitter to approximately £91.

Importantly, systems such as MiQuit could be particularly useful for the high proportion of pregnant smokers who currently do not access ‘traditional’ methods of support 45, 46. For example, in the United Kingdom approximately 83% of pregnant smokers do not use support offered 46 but, if encouraged, many of these may use text support.

### Strengths and limitations of the study

A limitation is that this RCT did not have a specified primary outcome; however, although multiple cessation outcomes were used, we indicated a priori which was anticipated to be the most appropriate as a primary outcome (outcome 1) 19. Consequently, as we demonstrated that outcome 1 was feasible to measure, it is reasonable to use these data to represent MiQuit's probable treatment effect. However, caveats to the interpretation of non‐primary RCT outcomes still apply. Additionally, completeness of follow‐up and biochemical validation rates were not optimal, potentially reducing statistical power. However, we assumed conservatively that women lost to follow‐up were still smoking 27 and outcome ascertainment rates were slightly higher in the usual care group; both factors would tend to attenuate rather than inflate the observed intervention effect. Consistent with this observation, the complete case analyses showed stronger intervention effects for most smoking outcomes, including a statistically significant between‐group difference for cessation outcome 1. As with many RCTs, a further limitation is the unknown generalizability of findings to all pregnant smokers. We did not record data systematically on the numbers or characteristics of pregnant smokers attending hospital units during trial recruitment, so we cannot say how representative the trial sample is although, based on socio‐economic characteristics and smoking rates at pre‐pregnancy and baseline, the sample was generally representative of women who smoke in pregnancy and are recruited to trials 10. Ease of recruitment in antenatal care settings suggests that there is a substantial cohort of pregnant smokers who would be likely to use MiQuit if offered this as part of routine care. Moreover, we have already shown that 3–4% of pregnant smokers will initiate MiQuit after receiving a one‐page leaflet advertising this in their ‘antenatal booking pack’ 22.

A key strength is that this is the largest RCT to investigate the efficacy of text message‐based, self‐help cessation support which is appropriate for and can be followed safely by pregnant smokers. The study was conducted to the highest RCT standards; it employed remote randomization, those enrolling participants were blind to treatment allocations and abstinence was biochemically validated. Additionally, researchers collecting outcome data were, where possible, blind to treatment allocations, so outcome ascertainment bias was minimized. Intervention fidelity was high, 98% of MiQuit recipients recalled receiving text message support, and similarities between adjusted and unadjusted analysis models imply that chance differences in groups’ baseline characteristics do not explain findings. Similarly, it seems unlikely that use of other ‘non‐trial’ cessation support explains findings; use of such support was very similar in both groups, except that usual care group women were more likely to report having discussed smoking with a midwife. Such support would be expected to increase cessation in the usual care group, reducing the apparent efficacy of MiQuit. Overall, therefore, it seems likely that differences between groups’ smoking rates are due to MiQuit and not to other factors.

## Conclusions

MiQuit is likely to be an effective smoking cessation intervention, and further evaluative research is needed. If further research is confirmatory, pregnancy‐orientated text message systems such as MiQuit could be made available quickly and cheaply alongside other first‐line support options to help pregnant smokers to stop.

### Trial registration

ClinicalTrials.gov NCT02043509. Registered 14 January 2014.

### Declaration of interests

None.

## Supporting information


**Table S1** MiQuit treatment effect estimates on seven smoking outcomes: comparison of baseline model (1) with models additionally adjusting for (2) heaviness of smoking, (3) partner's smoking status and (4) education (all missing = smoking).
**Table S2** MiQuit treatment effect estimates on seven smoking outcomes: comparison of missing = smoking and complete case analyses.

**Appendix S1** Standard NHS booklet on smoking cessation provided to all participants (‘baby on the way, quit today’).

**Appendix S2** Tailoring characteristics and examples of MiQuit text messages.
