# Supplementary material for: Large multi‐centre pilot randomized controlled trial testing a low‐cost, tailored, self‐help smoking cessation text message intervention for pregnant smokers (MiQuit)
Source: Addiction. 2017 May 2;112(7):1238–49. doi: 10.1111/add.13802 (PMC5488183; doi:10.1111/add.13802)
Supplement: Supplementary file 1 — Table S1 MiQuit treatment effect estimates on seven smoking outcomes: comparison of baseline model (1) with models additionally adjusting for (2) heaviness of smoking, (3) partner's smoking status and (4) education (all missing = smoking). Table S2 MiQuit treatment effect estimates on seven smoking outcomes: comparison of missing = smoking and complete case analyses. [file ADD-112-1238-s001.docx]

Supplementary Tables for Naughton et al “*Multicentre, randomised controlled trial testing a low-cost, tailored, self-help smoking cessation text message intervention for pregnant smokers (MiQuit)”*

**Table S1** MiQuit treatment effect estimates on seven smoking outcomes: comparison of baseline model (1) with models additionally adjusting for (2) heaviness of smoking, (3) partner’s smoking status and (4) education (all missing=smoking)

**Table S2** MiQuit treatment effect estimates on seven smoking outcomes: comparison of missing=smoking and complete case analyses
