# Supplementary material for: Large multi‐centre pilot randomized controlled trial testing a low‐cost, tailored, self‐help smoking cessation text message intervention for pregnant smokers (MiQuit)
Source: Addiction. 2017 May 2;112(7):1238–49. doi: 10.1111/add.13802 (PMC5488183; doi:10.1111/add.13802)
Supplement: Supplementary file 3 — Appendix S2 Tailoring characteristics and examples of MiQuit text messages. [file ADD-112-1238-s003.docx]

**Appendix 2 – Tailoring characteristics and examples of MiQuit text messages**

**14 tailoring characteristics used to individually tailor MiQuit text messages**

1. Motivation to quit (seriously planning to quit within the next 2 weeks, 30 days, 3 months or not planning to quit)
2. Whether quit date has been set (date of that quit date)
3. Most difficult situation to resist smoking (socialising, first thing in the morning, when stressed, during an urge)
4. Self-efficacy/confidence to quit smoking for the remainder of pregnancy (1 – 5 scale, 1 = not at all, 5 = extremely)
5. Number of cigarettes smoked per day (from the Heaviness of Smoking Index)
6. Time to first cigarette (from the Heaviness of Smoking Index)
7. Has a partner that smokes
8. Longest previous quit attempt (less than 2 weeks, 2-5 weeks, 6-11 weeks, 12 weeks + or not previously attempted to quit)
9. Difficulty of longest previous quit attempt (1 – 5 scale, 1 = not at all, 5 = extremely)
10. Perceived main disadvantage of quitting (increased tension, weight gain, feeling dull/bored, none of these)
11. Perceived main reason for quitting, apart from for baby’s health (your health, taking control, saving money, none of these)
12. Determination to quit smoking for good (1 – 5 scale, 1 = not at all, 5 = extremely)
13. Gestation
14. Smoking status at 3 and 7 weeks into programme

**Example text messages**

*Day in delivery schedule and tailoring characteristics used for each message listed in parentheses*

**Motivation**

(Day 7: *Quit date set but not passed, smokes 5 or less per day*)

“Even smoking [*smoking rate*] a day can have a big impact on your baby's health. So it's great news that you've picked a quit date and plan to avoid this”

(Day 19: *low motivation, most important reason for quitting is money, baseline smoking rate 11-15 per day*)

"To boost your motivation, keep reminding yourself why quitting is important to you - to have a healthy baby and an extra £30 a week to spend"

**Preparation**

(Day 31: *Low motivation, not set a quit date, most difficult situation is socialising*)

“If you're going to set a quit date [*name*], choose a time when you're least around other smokers e.g. if you socialise more on weekends, quit on a Monday”

(Day 33: *high motivation, most difficult situation is morning, partner smokes*)

"If you struggle to not smoke in the mornings, work on changing your morning routine, try changing the order you do things & ask others not to smoke near you"

**Self-efficacy**

(Day 37: *low self-efficacy, low determination to quit for good*)

“Hi [*name*] hope you're well & feeling a bit more determined to quit. You really can do this. Let yourself become more confident each time you resist smoking”

(Day 16: *Not set a quit date, longest previous quit less than 6 weeks, previous quit attempt was difficult*)

"Don't let your past quitting experience put you off trying to quit this time. Think about which situations were the hardest & make a plan to overcome them"

**Outcome** **expectancies**

(Day 57-59 depending on track: *Not abstinent at 3 weeks into programme, abstinent at 7 weeks into programme*)

"You should find your recent quit attempt got easier when you went over the 2 week point. Withdrawal symptoms should be fading & cravings to smoke less common"

(Day 18: *low motivation, main disadvantage with quitting is tension*)

"You are concerned about dealing with tension & irritability when quitting. Taking some time-out or trying mild exercise can boost your mood & help you relax"

**Relapse** **prevention**

(Day 52: *Abstinent at 3 weeks into programme, not abstinent at 7 weeks into programme*)

“What situation brought you back to smoking during your recent quit attempt? Write down some things you could do next time to help manage your cravings”

(Day 22: not tailored)

"Text QUIZ to this number if you need a brief distraction from a craving. Use QUIZ as much as you like, but it works best if you agree not to smoke when using it"

**Postpartum**

(Day 78: *high determination to quit long term, abstinent at 7 weeks into programme, most difficult situation is socialising*

"You may find after giving birth that some situations are harder at first - watch out for social situations. Rehearse saying 'no thanks, I've quit'"

(Day 67-69 depending on track: *main disadvantage with quitting is* *weight gain*)

"You are concerned about weight gain from quitting long term. However, weight gain is preventable - healthy snacks between meals and keeping active works :-)"

**Baby information**

(Sent at 20 weeks gestation)

“Baby info: Week 20 - your baby is about the length of a banana! They are probably quite active at the moment - they can even do the occasional somersault!”

**Additional support if quit date set**

(Sent day before quit day)

"Tomorrow's the big day. Quitting will be tough but you can do it - we believe in you :-) Just take each day as it comes and remember - not even a puff"

**HELP instant support**

"Remember that cravings only usually last a few minutes at a time. Keep yourself busy or distracted and they WILL pass."

**SLIP instant support**

"Think about what made you light up? How could you prevent it next time? A good way can be to remind yourself why you are quitting"
